# Fast Degradation of Azo Dyes by In Situ Mg-Zn-Ca-Sr Metallic Glass Matrix Composite

**DOI:** 10.3390/ma16062201

**Published:** 2023-03-09

**Authors:** Rui Jin, Gaojiong Wang, Xin Wang, Wei Yang, Yumin Qi

**Affiliations:** Hebei Key Laboratory of New Functional Materials, School of Material Science and Engineering, Hebei University of Technology, No. 5340, Xiping Road, Beichen District, Tianjin 300401, China

**Keywords:** metallic glass matrix composite, magnesium, in situ composite, azo dye degradation

## Abstract

Mg-based metallic glass (MG) has attracted extensive attention in the field of wastewater treatment due to its high decolorization rate in degrading azo dyes. However, the azo dye degradation rate of Mg-based MGs is strongly dependent on the particle size. Improving the intrinsic degradation efficiency using large particles is of great interest for future applications. In this work, in-situ metallic glass matrix composites (MGMCs) with high Mg content were successfully prepared by melt spinning. It is found that when the Mg content is 79–82%, the as-spun sample shows typical glassy characteristics. The SEM and XRD tests confirm that the as-spun sample is composed of α-Mg dendrite, multiple Mg-Zn intermetallic particles and an MG matrix. The degradation experiment using Direct Blue 6 and a 500 μm particle sample demonstrate that the Mg_82_Zn_14_Ca_3_Sr_1_ MGMC sample degrades azo dyes faster than typical Mg-Zn-Ca MG alloy. It can be attributed to the galvanic cell effect on the α-Mg/MG interface, which reduces the waste of active Mg atoms in the MG matrix according to the corrosion protection mechanism by the α-Mg anode sacrifice. This result provides a new perspective and insight into the design of azo dye degradation alloys and the understanding of degradation mechanisms.

## 1. Introduction

Mg-based metallic glass (MG) has attracted new research interests in the field of wastewater treatment due to its excellent performance in removing azo dyes and heavy metal ions from aqueous solutions [1,2]. It is well known that azo dyes are toxic to aquatic life and mutagenic for humans, and they are difficult to degrade completely in natural water because of the presence of a stable chemical bond -N=N- [3]. Therefore, azo dye pollution has been regarded as a stubborn environmental pollution factor that has threatened human health for a long time. At present, the effective treatment of azo dye wastewater depends on complex conditions, expensive equipment or high energy consumption [3,4]. It is difficult to obtain the ideal treatment effect (decolorization) at low costs, which forces printing and dyeing enterprises to stop using azo dyes. However, the threat of azo dye molecules accumulated in natural water to the ecological environment cannot be ignored. In recent years, it has been found that the environment-friendly Mg-Zn-Ca MG alloy composed of non-toxic metallic elements can rapidly degrade azo dyes without adding other chemicals, such as hydrogen peroxide [5], providing a new choice for treating azo dye pollution existing in natural water bodies.

Mg-based bulk metallic glasses (BMGs) are usually strong, corrosion-resistant and very brittle due to their homogeneous single-phase structure and disordered atomic arrangement [6,7]. Due to the aim of improving the application potential of Mg-based BMGs as structural materials, it has been a hot research topic to design metallic glass matrix composites (MGMCs) to adjust deformation behavior and improve plasticity [8,9,10]. On the other hand, Mg-based MG materials also show good potential for functional applications without regard to the mechanical properties. In 2012, Wang et al. reported for the first time that the azo dye degradation performance of Mg-Zn-Ca MG alloys was much higher than that of commercial crystalline Fe powders [5]. The high resistance of the glassy structure to water corrosion reduces the waste of zero-valent Mg (Mg^0^), thus increasing the proportion of active metal actually participating in the degradation reaction. Subsequently, the research of Mg-Zn-Ca MG alloys in the treatment of azo dye wastewater has attracted more attention, and relevant reports mainly focus on the following aspects: (1) low-cost preparation methods; (2) optimization of alloy composition; (3) the influence of second-phases or partial crystallized precipitates; (4) surface treatment.

As with most azo dye degradation catalysts [11,12,13], the particle size of Mg-Zn-Ca MGs is an important factor affecting the degradation efficiency of azo dyes [14]. For example, when the particle size of Mg-Zn-Ca MG after ball milling is smaller than 20 μm, the larger specific surface area enhances the physical adsorption and increases the reaction site, resulting in complete decolorization of dye wastewater within 10 min [5]. However, Iqbal et al. used ribbon samples of similar Mg-Zn-Ca alloy to degrade azo dyes and found that it took several hours for the fully glassy ribbon sample to achieve full decolorization [15]. Likewise, Zhao et al. prepared Mg-Zn-Ca MG powder by gas atomization and found that the azo dye degradation performance of MG powder was significantly enhanced with the decrease in particle size (size effect) [14]. As a result, they found that the complete decolorization time of Mg_66_Zn_29_Ca_5_ MG was less than 2 min when the particle size was smaller than 25 μm [14]. In our opinion, the size effect should be sufficiently considered in the degradation performance test of Mg-Zn-Ca MG alloys, which may be the main reason for the difference in the performance results of similar material systems.

The phase composition of Mg-based MG alloys containing crystalline inclusions, including the crystal structure, volume fraction and size of the second phase, is also an important factor affecting the efficiency of sewage treatment. It is well known that alloy composition, cooling rate, mold surface, etc., will affect the precipitation behavior of the second phase from the glass-forming liquid before it freezes into a solid state [16]. For as-spun Mg-Zn-Ca MGs, it is reported that a sample with a partly amorphous structure containing some crystalline phases had a worse azo dye degradation performance than the fully glassy sample [15]. However, Ramya et al. synthesized partially glassy Mg-Zn-Ca alloy powder by one-step ball milling and proved that Mg-Zn-Ca amorphous/crystalline composite powder showed excellent azo dye degradation efficiency [17]. Chen et al. prepared MgZn alloys with full crystalline, semi-amorphous and full amorphous structures and found that the second phase had a significant impact on the dye degradation behavior of the ribbon [18]. It can be seen that whether the formation of the crystalline second phase in Mg-Zn-Ca systems is conducive to the rapid degradation of azo dyes needs further study.

By controlling the corrosion process of Mg-based MG alloy, especially through surface treatment, the azo dye degradation efficiency of MG alloy can be effectively improved. Luo et al. prepared nano-porous copper on the surface of ball-milled Mg_65_Cu_25_Y_10_ MG powder by dealloying, which not only increased the specific surface area, but also accelerated the dissolution of active atoms through a local Galvani cell reaction, thus improving the azo dye degradation efficiency [19]. Chen et al. found that the addition of NaCl can improve the degradation rate of the Mg-Zn-Ca MG ribbon by accelerating the surface corrosion coarsening and the formation of ZnO-like nanosheets in the later stage [20]. Recently, Chen et al. treated Mg-Zn-Ca MG ribbons using a citric acid solution to form a porous surface, together with the Zn/ZnO crystalline phase and found that the azo dye degradation property was significantly enhanced by delivering more primary hydrogen [H] and $O_{2}^{•-}$ radicals to Mg [21]. Similarly, the Mg-Cu-Ag-Y MG ribbons after dealloying treatment also improved the catalytic degradation efficiency of the pesticide cis-cyanthrin through the nano-porous network [22]. Zhang et al. studied the azo dye degradation behavior of the Mg_63_Cu_16.8_Ag_11.2_Er_9_ MG ribbon and pointed out that the degradation process of dye molecules includes physical adsorption and a reduction/degradation reaction (Mg^0^ − 2e^−^ = Mg^2+^), and the efficient physical adsorption on the surface of an MG alloy plays a dominant role in the early stage of azo dye degradation [23].

Mg-Zn-Ca BMG is a lightweight alloy composed of all non-toxic elements [24], which is very commendable for environmentally friendly sewage treatment. Therefore, it is important to adjust the composition of the alloy without changing the main elements to improve the intrinsic degradation performance for azo dyes. Ren et al. investigated the effect of Mg content in Mg-Zn-Ca MGs on azo dye degradation performance and found that high Mg content is beneficial for improving the degradation efficiency because samples with high Mg concentration can provide more Mg^0^ required for the degradation reaction of azo dyes [25]. However, when the Mg content exceeds 75% (in at.), the Mg-Zn-Ca alloy cannot maintain a fully amorphous structure [25]. This is not consistent with the early strategy of obtaining a fully glassy structure for designing an azo dye degradation alloy, so the azo dye degradation behavior of the high Mg content alloy has not been further studied. In this work, we designed several in-situ MgZn-based MGMCs with high Mg content of more than 79% (in at.) based on an Mg_66_Zn_30_Ca_3_Sr_1_ alloy, which has the highest glass-forming ability (GFA) up to date [26]. We analyzed the glassy structure characteristics using XRD and DSC and investigated the azo dye degradation behavior and decolorization mechanism of the sample with a high Mg content of up to 82%.

## 2. Materials and Methods

### 2.1. Materials Preparation

Raw materials with purities of Mg 99.9%, Zn 99.99%, Ca 99% and Sr 99.9% (in wt.) were used to prepare the master alloys with the nominal composition Mg_66+x_Zn_30−x_Ca_3_Sr_1_ (x = 13, 14, 15 and 16, in at.%). All alloys were weighed by using a precision balance (Mettler, ME204E, Columbus, OH, USA) with an accuracy of 0.0001 g. The weighed raw materials were put into quartz cups in a vacuum furnace with an ultimate vacuum of about 1 × 10^−3^ Pa, and induction heating was used to melt the alloys with Ar protection. The melted liquid was poured into the copper mold to obtain master alloy ingots, which were cut into pieces with pliers. The master alloy pieces were put into a quartz tube with a round hole (1 mm diameter) at the bottom, which was put in a spinning furnace (Shanghai MTINST, SDM-0.02, Shanghai, China) with Ar protection for re-melting. The melted liquid was blown onto a running copper rod with a rotating speed of 2000–3000 rpm through Ar to prepare ribbons with a width of 5–10 mm. For the confirmation of composition, an energy dispersive spectrometer (EDS) was performed, and the results are shown in Table 1. It shows that the actual composition of the sample prepared in this paper is similar to the nominal composition, which can be used as a model material to study the effect of Mg content on the azo dye degradation behavior. The as-spun ribbons were ground into a powder with scissors and mortar. Stainless steel sieves with 80-mesh and 100-mesh holes were used to screen all the powder samples to avoid the influence of particle size difference on the subsequent experimental results. For the convenience of description, Mg_66+x_Zn_30−x_Ca_3_Sr_1_ (x = 13, 14, 15 and 16) alloy powder samples are simplified as Mg79, Mg80, Mg81 and Mg82 according to the content of Mg.

### 2.2. Material Characterization

An X-ray diffractometer (XRD, Bruker D8 Advance, Cu Kα radiation, Billerica, MA, USA) was used to examine the phase composition of powder samples with a scanning speed of 8°/min and a scanning angle range of 10–90°. A differential scanning calorimeter (DSC, Setaram Themys ONE, Cranbury, NJ, USA) was used to measure the glass transition characteristics and thermal stability of the as-prepared samples under N_2_ protection at a heating rate of 20 °C/min. A scanning electron microscope (SEM, Hitachi S-4800, Tokyo, Japan) was used to examine the microstructure of the ribbon samples and the surface micromorphology of the particles after azo dye degradation experiments. An energy dispersive spectrometer (EDS) was used to examine the micro-area chemical composition. Image Pro Plus software was used to carry out particle size statistical analysis based on the optical photos taken by a metallurgical microscope (Zeiss, Axio Imager M2m, Jena, Germany). X-ray photoelectron spectroscopy (XPS, Thermo Scientific, Waltham, MA, USA, K-Alpha, with Al Kα X-ray monochromator) was used to analyze the electronic states of various elements on the surface of samples after azo dye degradation.

### 2.3. Azo Dye Degradation Test

Commercial direct blue 6 powder (DB6, C_32_H_20_N_6_Na_4_O_14_S_4_) and deionized water were mixed at a ratio of 0.2 g/L. In order to fully dissolve DB6 molecules, the mixture was stirred for 1 h with a magnetic stirrer at room temperature. For each degradation experiment, 0.2 g of alloy powder was put into 50 mL of azo dye simulation solution, and the mixture was kept in a water bath at 25 °C with magnetic stirring. After stirring for a certain time (0.5, 1, 2, 5, 10, 20, 40, 60 min), a syringe was used to extract about 0.5 mL of the treated solution and pass it through the membrane filter (nylon 66, 0.45 μm diameter pore) for filtering. The degraded liquid was injected into the quartz cuvette after different times, and a UV-visible spectrophotometer (UV, Shanghai Yuanxi UV-6000, Shanghai, China, wavelength scanning mode, tungsten lamp light source, wavelength range of 400–800 nm, scanning speed interval of 1 nm, scanning accuracy of 10°) was used to test the absorbance of different solutions. In order to recover the powder sample after each degradation test, the mix solution was filtered with a medium-speed filter paper with a pore diameter of 30–50 μm, and then the obtained wet powder was dried in a vacuum drying oven (Tianjin Taist, Tianjin, China, DZ-1BC II) at 102 °C for 1 h.

## 3. Results

### 3.1. Microstructure

**Figure 1:**
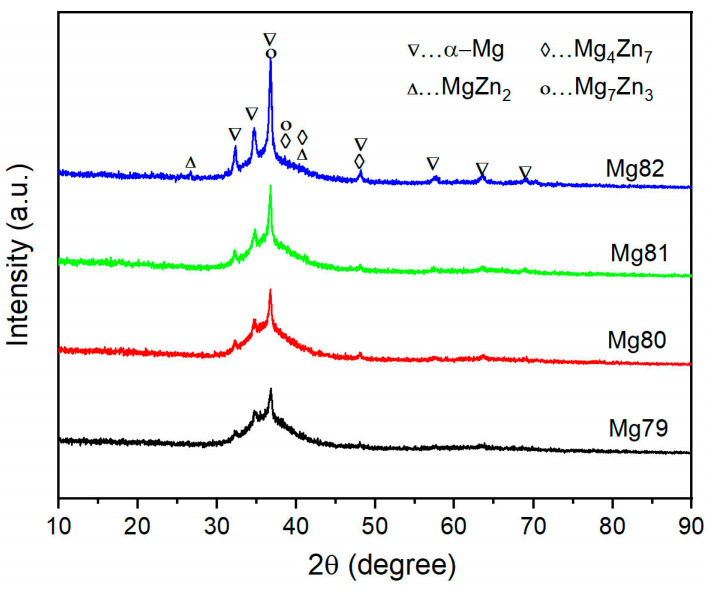
XRD patterns of Mg-Zn-Ca-Sr MGMC samples with different Mg content.

Figure 1 shows the XRD patterns of the as-prepared samples. All patterns are composed of a diffuse peak and several sharp crystalline phase diffraction peaks. The diffuse peak comes from the amorphous structure, indicating that all samples contain a certain amount of amorphous phase. The crystalline diffraction peaks can be divided into two groups: Mg and Mg-Zn intermetallic. For the Mg groups, the diffraction peak angle is highly consistent with the primary α-Mg dendrite phase in reported Mg-based MGMCs [27,28]. It is suggested that the as-prepared samples with different Mg content all contain α-Mg dendrite. The intensity of α-Mg peaks in the XRD pattern increases with the increase in Mg content, indicating that the addition amount of Mg significantly affects the volume fraction of the primary α-Mg crystalline phase. For the Mg-Zn intermetallic compounds, the diffraction peaks are much lower, which are generally hidden and interfered with by background noise. Furthermore, the common Zn-rich intermetallic compounds in the Mg-Zn alloy system, such as Mg_7_Zn_3_, Mg_4_Zn_7_ and MgZn_2_, have similar diffraction peak angles, so it is difficult to accurately distinguish them through the XRD patterns in this work. According to the reported XRD analysis of Mg-Zn phases in the literature [29,30,31,32], it can be inferred that the intermetallic phase in the Mg79-Mg82 sample may be a mixture of Mg_7_Zn_3_, Mg_4_Zn_7_ and MgZn_2_ phases. In addition, the diffraction peak of α-Mg in sample M82 is significantly higher than that of Mg_−_Zn compounds, which implies that the amount of crystalline phase α-Mg is significantly higher than the other crystalline phases.

**Figure 2:**
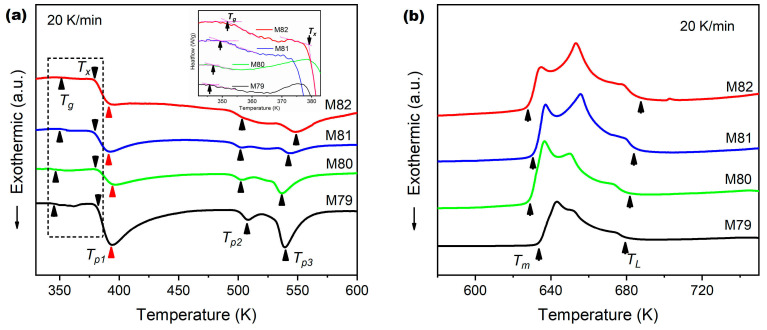
DSC curves of Mg-Zn-Ca-Sr MGMC samples with different Mg content showing: (**a**) low temperature range for glass transition and crystallization, with the inset showing a partially enlarged view of the temperature range marked by the dotted box; (**b**) high temperature range curves showing the melting of different samples.

In order to further confirm the presence of the MG phase, the DSC test is performed on the powder samples. The inset of Figure 2a shows a partially enlarged view of the low temperature range marked by the dotted box, which indicates that all samples have the characteristics of a glass transition at about 350 K. It is suggested that there is a certain amount of glassy phase in the samples, which is in good agreement with the XRD result shown in Figure 1. When the temperature rises above 370 K, three exothermic peaks appear on the DSC curves of the four samples, which are labeled as T_p1_, T_p2_ and T_p3_, as shown in Figure 2a, respectively. These exothermic events can be attributed to the complex crystallization transformation of the glass phase, which is also considered as a representative feature of the existence of the glassy phase [33,34].

The thermal parameters of as-prepared powder samples with different Mg content, including the glass transition temperature T_g_, the onset crystallization temperature T_x_, the three peak crystallization temperatures T_p1_, T_p2_, T_p3_, and the supercooled liquid region ΔT (T_g_ − T_x_), are measured and calculated from the DSC curves and listed in Table 2, respectively. It can be seen that T_g_ is slightly improved with the increase in Mg content, which implies that the MG phase in different MGMC samples shows different thermal stability. However, T_x_ has a decreasing trend with the increased Mg content. It may be induced by the increase in α-Mg/MG interface area (α-Mg volume fraction increases), which significantly increases the number of heterogeneous nucleation sites and induces crystallization at lower temperatures [35,36]. For the supercooled liquid region ΔT, it decreases obviously with the increase in Mg content, indicating that the glass-forming ability (GFA) of the Mg-Zn-Ca-Sr alloy decreases. This is because the increase in Mg content makes the alloy composition leave the optimal GFA composition range, resulting in the precipitation of crystalline phases. In addition, with the increase in Mg content, the area of the three crystallization exothermic peaks (relates to crystallization exothermic enthalpy) has a trend of gradually decreasing, which indicates that the amount of glassy phase in the MGMC samples gradually decreases.

**Figure 3:**
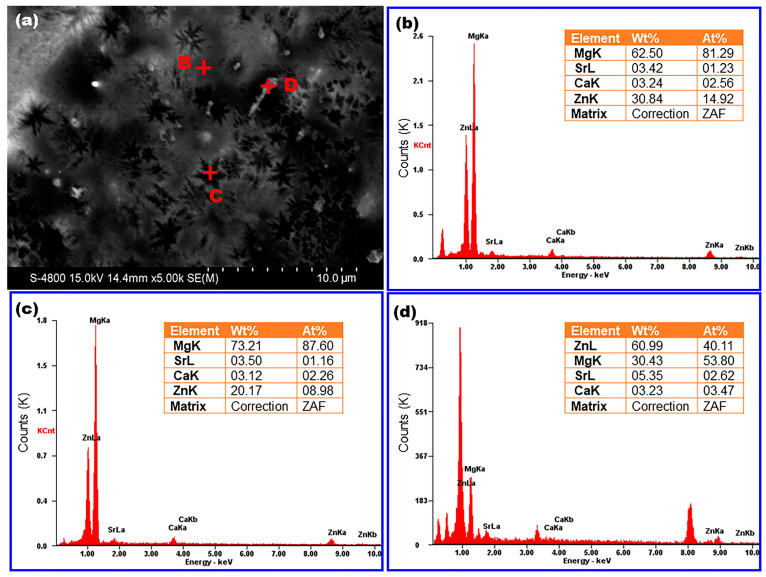
(**a**) Typical SEM image of an Mg_82_Zn_14_Ca_3_Sr_1_ MGMC sample; (**b**–**d**) EDS results of point B, C and D as marked in (**a**).

Figure 3a displays a typical SEM image of an as-spun Mg_82_Zn_14_Ca_3_Sr_1_ sample (Mg82), which shows that some snowflake-like patterns and white irregular particles are unevenly distributed on the gray matrix. It is very intuitive that the volume fraction of the snowflake phase is significantly higher than that of white particles. For further analysis, the EDS results of the gray matrix (point B), snowflake phase (point C), and white particles (point D) in Figure 3a are shown in Figure 3b–d. The Mg content in the snowflake area is significantly higher than that in the gray matrix and white particles, and it is also higher than the nominal composition of the M82 sample. It is well known that the composition of solvent elements in the solid solution phase can fluctuate in a large range, so the snowflake phase should be a solid solution phase with Mg as the solvent. In addition, the morphology of this snowflake structure is consistent with that of Mg dendrite in the literature [27]. Combined with the XRD results shown in Figure 1, it can be considered that the snowflake phase pattern comes from α-Mg dendrite crystals. For the white particles, the EDS results in Figure 3d show that the Mg:Zn atomic ratio is 53.8:40.1, which can only be achieved when the particles are composed of Mg-rich phase Mg_7_Zn_3_ and Zn-rich phase Mg_4_Zn_7_ or MgZn_2_ in a certain proportion. Therefore, it is suggested that the white particle phase in Figure 3a is a particle cluster composed of Mg_7_Zn_3_, Mg_4_Zn_7_ and MgZn_2_, which is consistent with the XRD test results shown in Figure 1. After excluding primary α-Mg and Mg-Zn composite particles, the gray matrix phase should be amorphous MG. To sum up, the M82 sample is a typical in situ formed Mg-based MGMC with the MG phase as the matrix and α-Mg dendrite and Mg-Zn compounds as the precipitation phases.

### 3.2. DB6 Degradation

**Figure 4:**
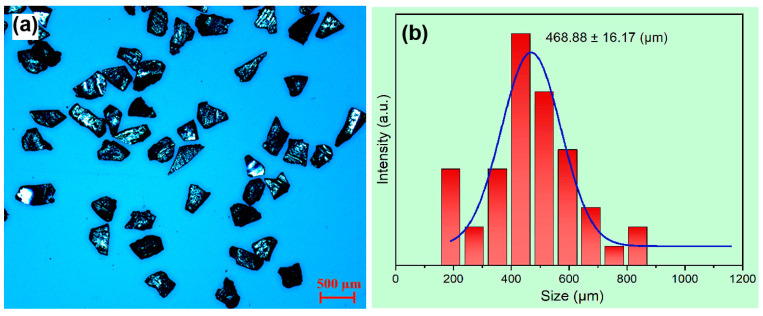
(**a**) Optical microscopic image of sample M82 power; (**b**) particle size statistical analysis of sample M82 using Image Pro plus 6.0 software.

The azo dye degradation efficiency of the MgZn-based MG alloy is significantly particle-size-dependent, according to the reports [14,17]. Therefore, particle size should be precisely measured before determining the azo dye degradation performance for any sample. Figure 4 shows the optical microscopic image and corresponding particle size statistical analysis of sample M82 after sieving between the 80-mesh sieve and the 100-mesh sieve. The particle size after screening fluctuates from ~200 to 850 μm, with an average value of 468.88 ± 16.17 μm, which is much larger than that of ball-milled [5] and gas-atomized [14]. In addition, the particles after grinding have sharp edges and corners, and their geometric shape shows the characteristics of irregular shape, which is consistent with the fracture of brittle materials. It is known that the properties of MG alloys and MGMC materials are obviously affected by various conditions such as thermal history [37,38], oxidation [39,40,41], and residual stress [42,43,44], etc. Relatively, large particles are less affected by the heating change, oxidation and other conditions due to low specific surface area. Therefore, the azo dye degradation performance tested with large particle samples is closer to its intrinsic degradation property than that of very small particle samples. The development of high-efficiency azo dye degradation materials with a large sample size is of great significance to the stability of the azo dye degradation performance and a long period of validity of sewage treatment agents.

**Figure 5:**
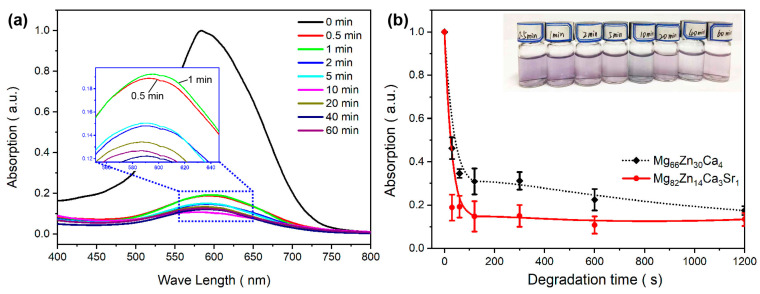
(**a**) Absorption spectra of the M82 sample after immersing in DB6 solution for different times; (**b**) comparison of the degradation curves of the Mg82 sample and Mg_66_Zn_30_Ca_4_ MG alloy under similar particle size, with an inset showing the macroscopic optical photos of the DB6 solution degraded by using M82 at different times.

Figure 5a shows the UV-visible absorption spectrum of the simulated dye wastewater solution, which is degraded with M82 powder at different times. The absorption spectrum of the DB6 solution usually has significant absorption between 500 and 700 nm, with a peak of about 570 nm [45,46]. After degradation for 0.5 min, the absorption peak height is significantly reduced by 84%, which shows the high efficiency of the M82 sample in the degradation of azo dyes. It can be seen from the locally enlarged image in Figure 5a that the difference between the absorption spectra of 0.5 and 1 min samples is not significant, indicating that DB6 dye molecules are mainly degraded or removed from the solution within 0.5 min. It can be verified from the macroscopic photo inserted in Figure 5b, which shows all degradation time solutions have a similar color of light purple.

Figure 5b shows the absorbance/degradation time curve (degradation curve) of the M82 sample and Mg_66_Zn_30_Ca_4_ MG (MZC) tested under the same conditions, such as degradation temperature, pH value, dosage, and particle size. In order to improve the comparability of data, all measured absorbance values (termed as A) are divided by that of the original DB6 solution for normalization. It clearly shows that the degradation curve of M82 is below MG and has a higher slope before 100 s, indicating that the azo-dye degradation rate of the M82 sample is higher than MZC. The results also show that the DB6 solution is significantly decolorized within 120 s, and the removal rate (R = (A_0_ − A_t_)/A_0_) is up to 85.2%, which is about 10% higher than that of the MZC sample (R = 77.5%) at the same time. Due to the fast degradation rate, the time window for manual operation is very small, and the sampling error is less than 60 s is large. Combined with the color change trend in the inset, the current results can prove that the azo dye degradation performance of M82 MGMC samples with high Mg content is better than that of the control sample MZC.

**Figure 6:**
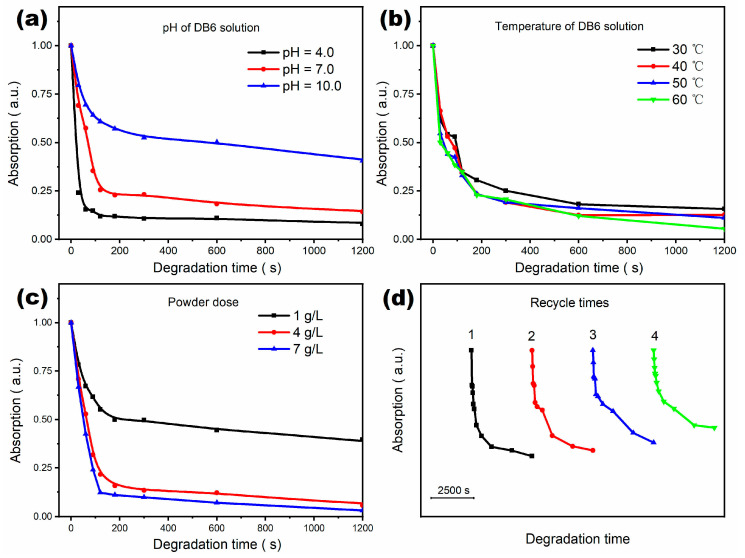
Degradation curves of Mg82 under different conditions of: (**a**) pH of DB6 solution; (**b**) treatment temperature; (**c**) dose; (**d**) recovery time.

Figure 6 shows the effects of pH value, degradation temperature, powder dose, and recycling times of powder on the azo dye degradation efficiency of M82 samples, respectively. It is found that the pH value significantly affects the azo dye degradation rate of M82 samples, as shown in Figure 6a. When the pH is 4.0, the removal rate of dye molecules at 120 s reaches ~90%, which is significantly higher than that under neutral conditions (pH = 7.0) and alkaline conditions (pH = 10.0). We know that Mg can generate a relatively stable Mg(OH)_2_ passivation film under a strongly alkaline environment. This film can block the contact between Mg^0^ in MGMC and DB6 molecules in solution, which is not conducive to the continuous degradation of azo dyes. Figure 6b shows the influence of degradation temperature on the degradation curve of the M82 sample. When the temperature of the DB6 solution changes from 30 to 60 °C, all the degradation curves are mixed together, showing similar degradation rates without any regularity. This result indicates that the effect of temperature on the azo dye degradation is not significant. It is suggested that the reaction between Mg-based MGMC and azo dyes may not be a thermal activation process. Figure 6c shows the effect of powder dose on the degradation curve of the M82 sample. Obviously, a higher dose can provide a higher specific surface area and more Mg^0^, which can improve the degradation rate of azo dyes. It is worth noting that when the dose exceeds 4 g/L, the difference between the degradation curves is small, indicating that high-speed degradation can achieve an economic balance between material consumption and degradation rate. Figure 6d shows the cyclic degradability of the recovered M82 powder. It can be clearly seen that after four recycles of M82 powder, the azo dye degradation efficiency is decreased in the early stage of the degradation experiment. The removal rate of DB6 molecule is only 25% at 120 s, but it can still reach 70% at 3600 s. It shows that the ability of M82 powder to rapidly degrade azo dyes is weakened after several recoveries. Although the recyclability and recycling rate of M82 cannot be comparable with that of a high-stability alloy (Cu/Fe/Co-based MG alloys [47,48,49]), it can still obtain potential applications in those situations without recycling, such as soil improvement, overall nitrogen removal of printing, and dyeing wastewater storage tank, etc. Benefiting from the degradability, lightness and non-toxicity of all component elements, Mg-Zn-Ca-Sr MGMC can degrade itself in nature without worrying about the secondary pollution of heavy metals contained in the sewage treatment agent.

**Figure 7:**
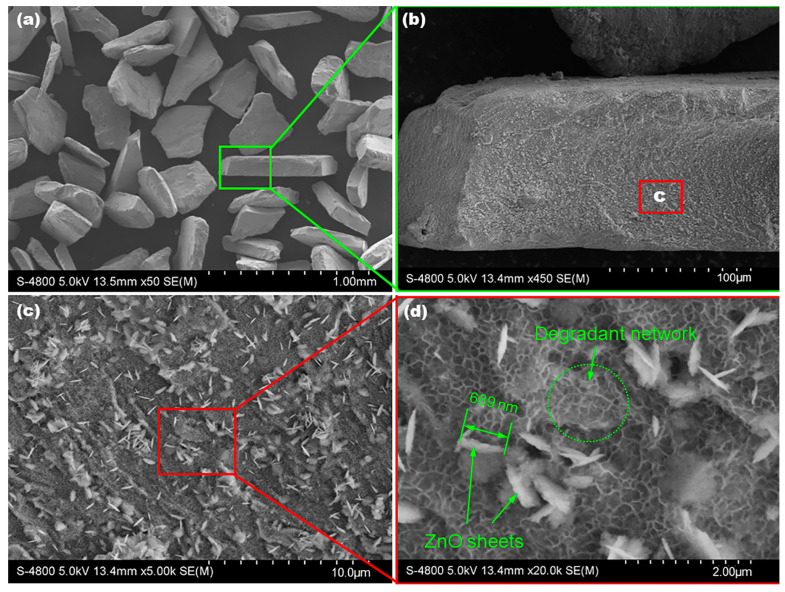
SEM images of the Mg_82_Zn_14_Ca_3_Sr_1_ MGMC sample after DB6 degradation for 60 min with different magnifications (**a**–**d**).

Figure 7 shows the typical SEM images of the Mg82 MGMC sample after DB6 degradation for 1 h with different magnifications. After reacting with the DB6 solution, the shape and size of the M82 particle have no obvious change, as shown in Figure 7a. It is implied that the sample powder has no obvious consumption in the degradation reaction. However, through the highly magnified SEM images displayed in Figure 7b–d, it can be clearly seen that there are significant changes to the surface morphology of the sample: (1) some white sheet products stand upright on the substrate (Figure 7c); (2) the surface is covered by a light gray network (Figure 7d). The white sheet products have a size of ~700 nm and a thickness of ~5 nm, which are generally considered to be ZnO or Zn(OH)_2_ deposits inferred from morphology. This is a common phenomenon in the azo dye degradation process of Mg-Zn-Ca MG alloy [5,20]. For the light gray network pattern, it can be found in different MG systems used for azo dye degradation [50,51,52], which is generally considered to be the degradation product of the reaction between dye molecules and the surface metal.

**Figure 8:**
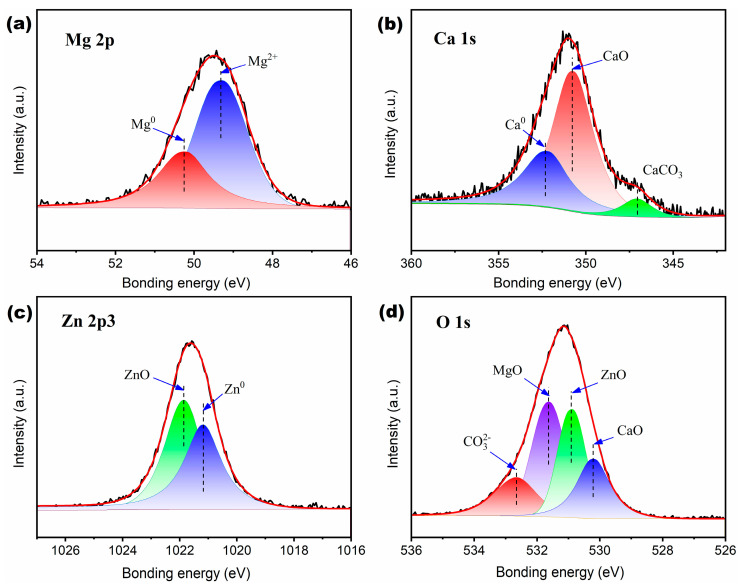
Partial peak analysis of local XPS spectrum of Mg82 sample after azo dye degradation: (**a**) Mg 2p; (**b**) Zn 2p; (**c**) Ca 1s and (**d**) O 1s.

Figure 8 shows the XPS spectrum analysis of the recovered M82 powder after the azo dye degradation experiment. Figure 8a shows that the Mg 2p peak can be decomposed into two peaks with different bonding energy of 49.3 and 50.2 eV, corresponding to Mg^2+^ and Mg^0^, respectively. The peak height of Mg^2+^ is about twice that of Mg^0^, which indicates that after soaking in DB6 solution for 1 h, Mg atoms on the surface of M82 particles form bivalent Mg deposits, such as MgO, Mg(OH)_2_ or MgCO_3_. It should be noted that the existence of the Mg^0^ peak implies that the surface metal of the particle still has the ability to degrade azo dyes, agreeing well with the results shown in Figure 6d. Figure 8b shows that the Ca 1s peak can be decomposed into three peaks with different bond energies, namely 347.0, 350.7 and 352.3 eV, corresponding to CaCO_3_, CaO and Ca^0^, respectively. It is very intuitive that the CaO peak is higher than CaCO_3_ and Ca^0^, which means that the sample surface contains a large amount of CaO. Considering that the XPS sample has undergone vacuum drying treatment, which may lead to the decomposition of hydroxide, these CaO may also come from the decomposition of Ca(OH)_2_. In addition, carbonate ions CO_3_^2−^ may come from the decomposition of DB6 molecules or the dissolution of CO_2_ in solution. Figure 8c shows that the Zn 2p^3^ peak can be decomposed into two peaks with similar bonding energies, 1021.2 and 1021.9 eV, corresponding to Zn^0^ and Zn^2+^, respectively. Compared with Mg and Ca, Zn is more stable in an aqueous solution, resulting in more Zn^0^ remaining on the surface of the M82 sample after the 1 h azo dye degradation test. Figure 8d shows that the O 1s peak can be decomposed into four peaks with different bonding energies, 530.2, 530.9, 531.6 and 532.6 eV, corresponding to CaO, ZnO, MgO and CO_3_^2−^ respectively. Among these peaks, the peak heights of MgO and ZnO are significantly higher than other peaks, indicating that the degradation products on the surface of the M82 sample are mainly composed of MgO and ZnO.

## 4. Discussion

The rapid azo dye degradation behavior of Mg-Zn-Ca MG has been attributed to the disordered atomic arrangement of its amorphous structure, which improves the corrosion resistance and reduces the corrosion waste of zero-valent magnesium (Mg^0^) [5]. Compared with Mg^0^ and Zn^0^ in crystalline alloys, zero-valent metals in MGs have a higher valence band and extended empty band structure, which can provide more active electrons for the degradation of azo dyes [53]. The electrons positioned at the conduction band can react with dissolved O_2_ to generate $O_{2}^{•-}$, accelerating the azo dye degradation [54,55,56]. However, the active zero-valent elements, such as Mg^0^, Zn^0^ and Ca^0^ contained in MGs, can react with water in nature, so the amorphous structure can only delay the corrosion in aqueous solution to a certain extent but cannot completely prevent the corrosion loss of them. In other words, when Mg-Zn-Ca MG is used to treat azo dye wastewater, a certain amount of active metal will inevitably be wasted due to the intrinsic corrosion. Therefore, how to protect the Mg-Zn-Ca MG alloy from corrosion and reduce the loss of active metal atoms has become one of the key scientific issues to improve the azo dye degradation efficiency.

In this work, we produce more Mg^0^ by increasing the Mg content in the alloy. Considering that Sr can improve the GFA of Mg-Zn-Ca MG alloys [26], we also introduce a small amount of Sr to reduce the adverse effect of Mg content change on the GFA of amorphous alloys. The results show that the Mg content of the MG matrix phase in Mg82 MGMC is effectively increased (Figure 3b), inducing improved azo dye degradation performance of the alloy. In addition, Mg-Zn-Ca-Sr MGMCs contain a small amount of Sr, which has a strong oxidation property. Therefore, it not only improves the GFA of the matrix phase but also helps to reduce the O content of the matrix alloy. Moreover, Sr and Ca have similar intrinsic properties and can react with water, resulting in an increase in [H] in the solution, which is also conducive to the degradation of azo dyes. However, the increase in Mg content eventually leads to the precipitation of crystalline α-Mg, which inevitably causes the reduction in glassy Mg atoms in the alloy. Interestingly, it is found that the azo dye degradation rate does not decrease due to α-Mg precipitation. The related mechanism can be understood by sacrificing anode protection.

**Figure 9:**
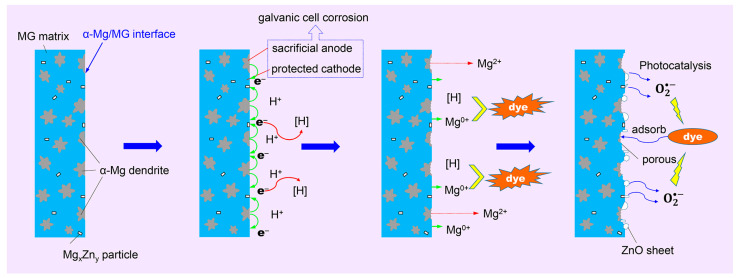
Schematic diagram showing the azo dye degradation mechanism by MgZn-based metallic glass composites with high Mg content.

It is well known that pure Mg and most of the Mg alloys can be used as anode materials to protect metal components from corrosion [57,58], according to the sacrificial anode theory. In our opinion, the α-Mg solid solution phase can play a key role in improving the azo dye degradation performance of the in situ MgZn-based MGMCs. Figure 9 shows the corresponding azo dye degradation mechanism based on the galvanic cell corrosion theory. The in situ MgZn-based MGMCs are composed of α-Mg, Zn-rich Mg-Zn intermetallic particles and MG matrix phase with high Mg content, in which the MG phase and α-Mg dendrite both have high volume fractions. When the composite sample is put into the DB6 solution, a small primary cell can be formed on the α-Mg/MG interface, and α-Mg dendrite acts as sacrificing anode with the reaction Mg − 2e^−^ → Mg^2+^. It forms a negative charge layer on the surface of the MG matrix, effectively preventing the discharge corrosion of MG as a cathode by sacrificing the α-Mg dendrite anode. In addition, the electrons e^−^ generated by the α-Mg dendrite combine with H^+^ to generate [H] [21], which diffuses into the solution and reacts with the dye molecules. As a result, azo dyes with a macromolecular structure are reduced and decomposed into small molecules.

In addition, MgZn-based MGMCs contain a certain amount of Zn-rich intermetallic particles, which may react with water as follows:

MgZn_2_ + 4H_2_O → Mg^2+^ + 2OH^−^ + 2H^2^↑ + Zn(OH)_2_↓ (1)

Mg_7_Zn_3_ + 20H_2_O → 7Mg^2+^ + 14OH^−^ + 10H^2^↑ + 3Zn(OH)_2_↓ (2)

Mg_4_Zn_7_ + 22H_2_O → 4Mg^2+^ + 8OH^−^ + 11H^2^↑ + 7Zn(OH)_2_↓ (3)

Through the chemical reaction revealed by Equations (1)–(3), Zn(OH)_2_ or ZnO-like nanosheets can be formed, as shown in Figure 7d, which is consistent with the research results in the literature [20,54,56]. These hydroxide/oxide particles in aqueous solution usually attract hydroxyl groups with a negative charge, which is beneficial to the formation of the free radical $O_{2}^{•-}$ and the degradation of azo dyes. Of course, when the degradation time is extended to a certain time, the MG matrix region can also form porous and smaller ZnO/MgO-like nanosheets, which are usually assembled into porous networks on the surface (Figure 6d). This porous network structure can increase the physical adsorption effect of the MGMC surface on dye molecules, improve the probability of zero-valent metals, [H] and $O_{2}^{•-}$ contacting dye molecules, and thus promote the degradation of azo dyes. Moreover, under the irradiation of natural light or ultraviolet light, these oxides with different scales can also induce luminescence catalysis and further degrade azo dye molecules.

In short, MgZn-based MGMC with α-Mg dendrite structure can degrade azo dyes through various mechanisms such as zero-valent metal, photocatalysis, etc. Its degradation process is doped with additional processes, including corrosion, sacrificial anode dissolution, etc. The azo dye degradation reaction mainly occurs in the local area of the MG matrix, and its size and shape depend on the morphology of the α-Mg dendrite. Therefore, a suitable dendrite obtained by controlling the morphology, size and proportion of α-Mg can affect the geometric distribution of the negative charge region, which is conducive to improving the azo dye degradation performance. In addition, the strategy of adjusting Mg content mainly uses cheap metal elements as raw materials, and common instruments and equipment are used in alloy melting and powder preparation, so the preparation cost of MGMC material is relatively low. Meanwhile, the process of using MGMC to degrade dye wastewater is simple, without heating treatment and adding other chemicals, and without special production lines or expensive equipment, which is conducive to reducing costs and has great significance to the promotion of catalysts.

## 5. Conclusions

(1) In situ Mg-Zn-Ca-Sr metallic glass matrix composites with high magnesium content were successfully prepared by melt spinning. SEM, DSC and XRD analyses confirm that the composite is composed of primary α-Mg dendrites, a metallic glass matrix and a few Mg-Zn intermetallic particles.
(2) In-situ Mg-Zn-Ca-Sr MGMCs with different Mg content have similar thermal stability. With the increase in Mg content, the glass transition temperature T_g_ and the melting point are increased, but the onset crystallization temperature T_x_ is decreased, resulting in the reduced supercooled liquid region. It indicates that the thermal stability and glass-forming ability of the MG matrix decrease.
(3) The Mg_82_Zn_14_Ca_3_Sr_1_ MGMC sample with a particle size of up to 500 μm shows a high azo dye degradation rate, indicating that the intrinsic azo dye degradation performance is improved by turning the Mg content. It is sensitive to pH during the treatment of azo dye wastewater, and the acidic environment is conducive to a high degradation rate.
(4) The rapid degradation of azo dyes can be attributed to the sacrificial anode protection induced by a crystalline Mg dendrite, which improves the utilization rate of zero-valent Mg in the MG matrix phase. It is suggested that the azo dye degradation performance of MgZn-based MGMC can be improved by regulating the microstructure of MGMC, which provides a new basis for the design of new dye wastewater treatment materials.

## Figures and Tables

**Table 1:** Compositions of as-spun samples with different Mg content determined by EDS.

Sample	Nominal Composition (at.%)	Tested Composition (at.%)				
		Mg	Zn	Ca	Sr	O
M79	Mg_79_Zn_17_Ca_3_Sr_1_	78.14	16.98	2.66	0.89	1.33
M80	Mg_80_Zn_16_Ca_3_Sr_1_	79.45	15.90	2.55	0.99	1.11
M81	Mg_81_Zn_15_Ca_3_Sr_1_	80.50	15.12	2.76	0.78	0.84
M82	Mg_82_Zn_14_Ca_3_Sr_1_	81.53	13.84	2.65	0.78	1.20

**Table 2:** Thermophysical parameters of as-prepared powder samples with different Mg content measured from DSC curves.

Sample	T_g_ (K)	T_x_ (K)	T_P1_ (K)	T_p2_ (K)	T_p3_ (K)	T_m_ (K)	T_L_ (K)	ΔT (K)
M79	346.6	382.6	394.1	507.1	539.4	632.1	679.2	36.0
M80	347.2	380.5	394.2	504.8	538.1	628.9	682.4	33.3
M81	349.9	379.6	389.9	503.7	543.8	629.8	686.1	29.7
M82	351.6	379.7	391.1	503.7	550.6	629.7	687.9	28.1

## Data Availability

Any further detailed data may be obtained from the authors upon a reasonable request.
